# Unilateral uveitis following adjuvanted Varicella-Zoster subunit vaccine in a patient with previously resolved Varicella Zoster acute retinal necrosis

**DOI:** 10.1186/s12348-025-00508-3

**Published:** 2025-08-02

**Authors:** A. Trinco, F. Zicarelli, F. Romano, M. Oldani, A. Riva, A. Invernizzi

**Affiliations:** 1 Department of Biomedical and Clinical Sciences, Ospedale Luigi Sacco, Eye Clinic, University of Milan, Via G.B. Grassi, 74, Milan, 20157 Italy; 2 Department of Biomedical and Clinical Sciences, Università degli Studi di Milano, Milan, Italy; 3 III Division of Infectious Diseases, ASST Fatebenefratelli Sacco, Luigi Sacco Hospital, Milan, 20157 Italy; 4 Save Sight Institute, University of Sidney, Sidney, NSW Australia

## Abstract

**Purpose:**

To report a case of intraocular inflammation following adjuvanted recombinant Varicella-Zoster subunit vaccine (RZV) in a patient with previously resolved VZV-related acute retinal necrosis (ARN).

**Methods:**

Single case report. Multimodal imaging, including true-color fundus photography, spectral-domain (SD-OCT) and dye-based angiography, was used to evaluate the retinal alterations.

**Results:**

A 73-year-old male, with past history of VZV-related acute retinal necrosis (ARN) developed unilateral cystoid macular edema and diffuse blood-retinal barrier (BRB) breakdown in the affected eye after receiving the second dose of RZV vaccine. A complete resolution of this complication was observed after a short course of systemic corticosteroids.

**Conclusion:**

Our case suggests a possible pathogen-specific intraocular inflammatory reaction, resembling immune reconstitution uveitis (IRU), following RZV administration in a patient with previously resolved VZV-related ARN. While RZV remains safe, intraocular inflammation should be considered as a possible side effect in patients with previously resolved VZV uveitis.

## Introduction

Acute retinal necrosis (ARN) is the most feared herpetic intraocular infection, commonly associated with Varicella Zoster Virus (VZV) or Herpes simplex virus (HSV) and characterized by centripetally progressing retinal necrosis, vitritis and occlusive arterial vasculitis [1, 2]. Considering its potential bilateral involvement, leading to complete permanent blindness, ARN diagnosis prompts for immediate intravitreal and/or systemic antiviral treatment [2, 3]. To prevent recurrences the treatment is usually continued for 6 months after the resolution of the acute phase of the disease [3].

Cases of ARN have been described, following the administration of live-attenuated vaccines, where the active substance is the weakened VZV, and were collectively explained as VZV recurrence or vaccine-induced reactivation, hence discouraging the use of this type of vaccine in patients with compromised immune systems or underlying pathologies [4–6].

The progressively increasing incidence of Herpes-Zoster, coupled with an elevated economic burden, has prompted the employment of new strategies for preventing disease recurrences [7, 8]. The recently licensed, adjuvanted recombinant zoster vaccine (RZV), combining Varicella-Zoster virus glycoprotein E (gE) with the AS01B adjuvant, has been welcomed by many regulatory authorities worldwide due to its high and long-lasting efficacy [9–17]. It consists of two doses administered 2–6 months apart and is now recommended by the European Medicines Agency (EMA) and by the U.S. Food and Drug Administration (FDA) for adults over 50 and in high-risk individuals aged 18 to 50, including immunocompromised hosts, considering its impossibility of causing VZV infection [18, 19].

Apart from minor side effects such as injection site pain, fatigue, fever, and myalgia commonly reported after RZV, overall safety has been established in registrative studies [17]. Nonetheless, potential local or systemic inflammatory events remain a subject of debate, with the eye being reported as a possible site of these adverse reactions [17].

With this paper, we present a case of intraocular inflammatory reaction following RZV administration in an eye with resolved VZV-related ARN, reporting for the first time a new possible complication and shedding light on its potential pathogenesis.

## Case report

A 73-year-old Caucasian male presented at our ophthalmologic emergency department with complaints of reduced visual acuity (VA), photophobia and tearing in the right eye (RE). Systemic hypertension and absence of a known immunocompromised status were noted during the medical history.

Upon examination, the RE visual acuity (VA) was 20/70 Snellen. Anterior chamber displayed marked perikeratic hyperemia, inferior granulomatous keratic precipitates, 2 + cells and irido-lenticular synechiae. The left eye (LE) VA was 20/20 with unremarkable anterior segment examination. Intraocular pressure was within normal limits bilaterally. Severe vitreous haze precluded a clear funduscopic examination in the RE, but distinguishable findings included blurred optic nerve head (ONH) margins, scattered hemorrhages at the posterior pole and in the superior retinal periphery, and a large yellow-whitish area involving the superior retina near the vascular arcade surrounded by smaller satellite areas in the superotemporal sector. The LE showed no signs of intraocular inflammation. Despite poor image quality due to vitritis, spectral-domain optical coherence tomography (SD-OCT) at the posterior pole revealed a preserved foveal anatomy and swollen ONH in the RE. OCT scans encompassing the affected areas in the superior peripheral retina revealed full-thickness retinal hyperreflectivity suggesting an ongoing retinal necrosis [20, 21].

Serology for *herpesviridae* was therefore performed and resulted positive for immunoglobulins G (IgG) for herpes simplex (HSV), VZV, and cytomegalovirus (CMV). Serology was negative for HIV and Quantiferon TB Gold test and treponemal pallidum particle agglutination assay (TPPA) and rapid plasma reagin (RPR) ruled out concurrent tuberculosis and syphilis, respectively. Aqueous humor tap followed by intravitreal injection of ganciclovir plus clindamycin was hence performed. Polymerase chain reaction (PCR) on aqueous sample detected elevated numbers of VZV DNA copies, confirming the viral etiology. Topical therapy with dexamethasone (q4 h), netilmicin (q6 h), and tropicamide (q8 h) was initiated to control anterior chamber inflammation, and the patient was admitted to the Infectious Diseases Department to commence systemic therapy with intravenous acyclovir therapy (750 mg tid). Progressive reduction of vitritis along with regression of necrotic areas was observed over the following two weeks, and the patient was eventually discharged with maintenance oral acyclovir therapy (400 mg bid).

**Fig. 1:**
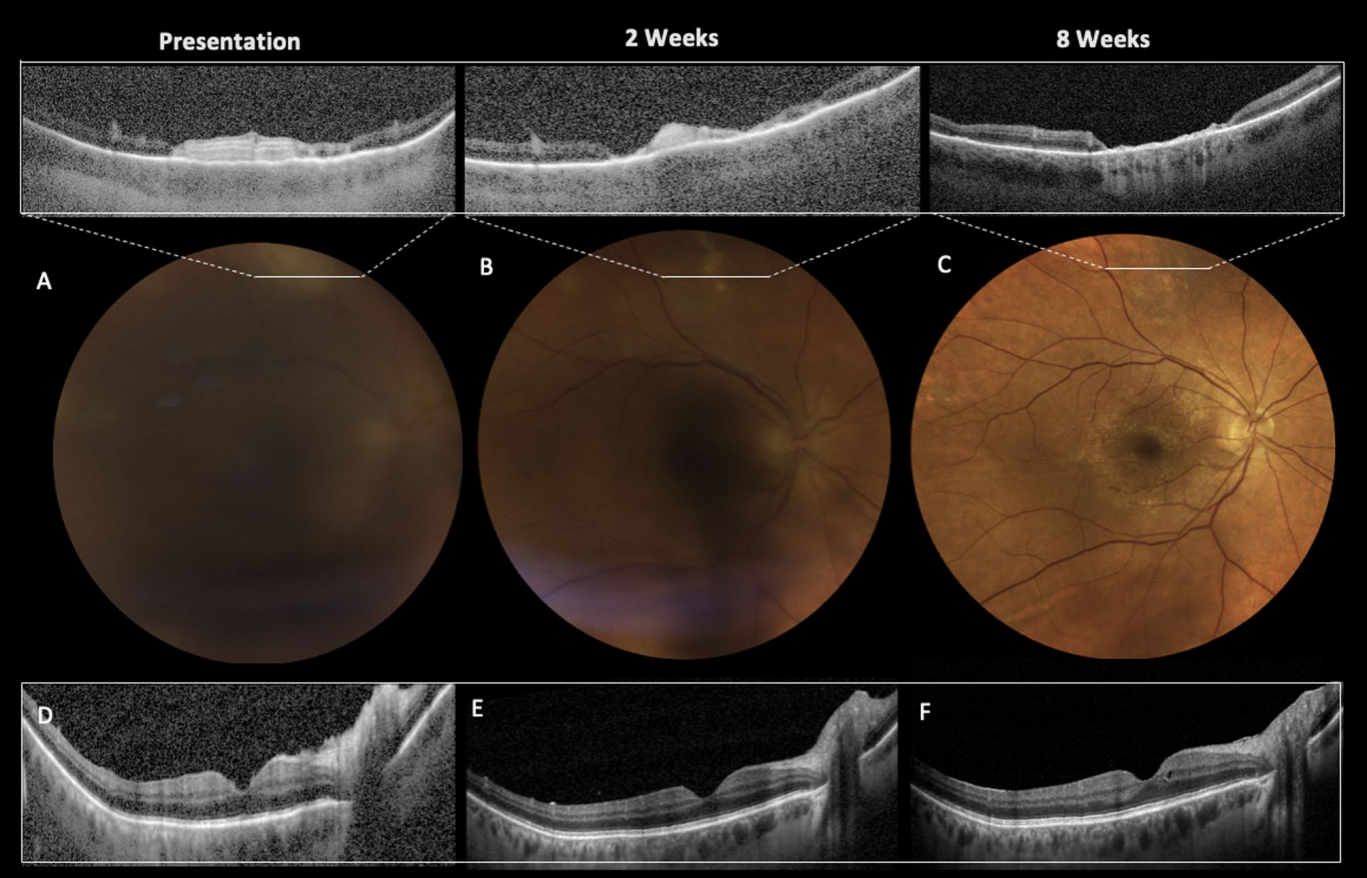
True-color fundus photograph and Spectral-domain OCT findings at presentation and post-therapy initiation. At presentation, an intense vitritis obscures retinochoroidal details, although the true-color fundus montage (**A**, EIDON, CenterVue, Padua, Italy) highlights blurred margins of the optic nerve head and multifocal white-yellowish lesions in the superior and superotemporal sectors. SD-OCT (Spectralis OCT2, Heidelberg Engineering, Heidelberg, Germany) reveals a preserved foveal shape and a mild optic nerve head edema (**D**). A peripheral OCT B-scan displays a full-thickness hyper-reflectivity of the retina localized above thickened choroid at the site of active retinitis (**A**, white-framed box). After initiation of the systemic therapy with acyclovir, progressive resolution of the vitritis (**B** and **C**) and of the optic nerve head edema (**E** and **F**) is observed. After 8 weeks of treatment, the necrotizing process completely regress, resulting in the formation of an atrophic scar involving all the retinal layers (**C**, white-framed box)

Two months later, BCVA had improved to 20/20 with minimal residual vitreous haze and atrophic scarring of the retinal lesions (Fig. 1). Following infectious diseases specialists recommendations RZV vaccination was scheduled to prevent recurrences, with doses administered six and eight months after the ARN presentation, respectively.

**Fig. 2:**
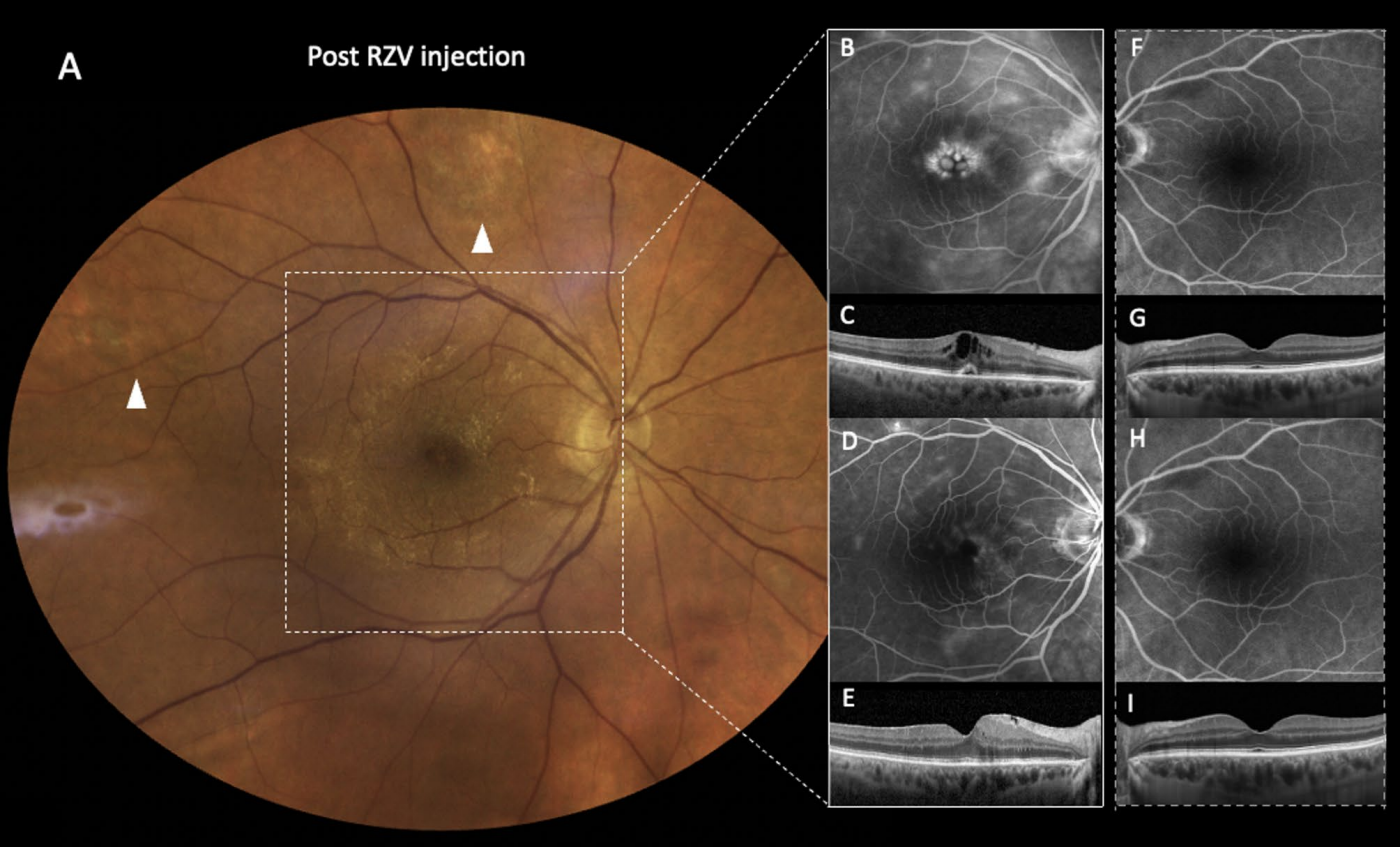
Multimodal imaging of the intraocular inflammatory reaction following the second dose of RZV. The true-color fundus photograph montage (EIDON, CenterVue, Padua, Italy) illustrates the right eye of the patient after the complete resolution of the VZV-related ARN and the administration of the second dose of RZV. A prominent atrophic scar is visible in the superior sector, accompanied by similar lesions in supero-temporal sector (**A**, white arrowheads) at the sites of resolved retinitis. No signs of vitritis or active necrosis are visible (**A**). Nevertheless, fluorescein angiography (HRA Heidelberg Engineering, Heidelberg, Germany) reveals an intense intraocular inflammation presenting with diffuse leakage around the optic nerve head (*hot disc*) and the vascular arcades and pooling of the dye in the macular area (**B**). SD-OCT confirms the development of a unilateral cystoid macular edema (**C**). After one month of systemic corticosteroid therapy a significant reduction in perivascular and peripapillary leakage is noted (**D**) along with complete resolution of the macular edema (**E**). Notably, no signs of inflammation can be observed in the left eye throughout the follow-up period (**F**-**I**)

One month after the second dose, the patient presented again to our Uveitis Service with unexpected VA worsening to 20/40 in the RE and unaffected VA in LE. While anterior segment examination did not show remarkable changes, SD-OCT demonstrated the development of unilateral cystoid macular edema (CME) in RE and absence of any relevant finding in LE. Fluorescein angiography confirmed the presence of intraocular inflammation revealing diffuse leakage around the optic nerve head (“hot disc”) and along the vascular arcades, indicative of blood-retinal barrier (BRB) breakdown. Progressive fluorescein pooling in the macular area highlighted the CME. No active foci of necrosis were detected, excluding a VZV reactivation and supporting the hypothesis of a reactive inflammatory process. After a week of ineffective topical therapy with dexamethasone drops, a systemic therapy with 25 mg oral prednisone daily was initiated. Progressive improvement of CME with complete resolution within a month confirmed our clinical suspect and prompted subsequent corticosteroid tapering and cessation with complete VA restoration (Fig. 2).

## Discussion

In this paper we report a case of unilateral intraocular inflammation and CME, developing after the two recommended doses of subunit VZV-vaccine in a patient with previously resolved VZV-related ARN, that was successfully treated with a short course of systemic corticosteroid therapy.

Extensive literature has reported intraocular inflammatory reactions, following various types of vaccination. Most documented cases involve anterior or posterior segment inflammation, often manifesting as multiple evanescent white dot syndrome (MEWDS), acute posterior multifocal placoid pigment epitheliopathy (APMPPE), or Vogt-Koyanagi-Harada (VKH)-like reactions. Typically, these cases showed a good response to corticosteroids, though occasionally required prolonged therapy [22–24].

Since FDA approval in 2017, the new adjuvanted recombinant vaccine against Varicella-Zoster (RZV), has progressively become the safest and most preferred preventive measure for VZV, due to its high and long-lasting efficacy and to the impossibility in causing VZV infection, even in immunocompromised patients [9, 11–17]. Therefore, the isolated reported cases of keratitis recurrence [25] and ARN [26] following vaccination with RZV could, collectively be explained with immunization failure rather than real vaccination-induced events. Nevertheless, the intense interest generated by RZV among ophthalmologists and other medical specialists, has warranted a close monitoring of other possible local and systemic inflammatory adverse effects (Aes) associated with RZV injection.

Two pro-inflammatory mechanisms of RZV that could explain what we observed in our patient can be hypothesized based on the existing literature. The first is a “pathogen-specific” reaction implying an effective immune response of the host against VZV after vaccination. In particular, humoral and cellular immunity induced by RZV are obtained through the synergy between the adjuvant AS01B, a potent initiator of the immune cell-mediated response, and recombinant VZV gE, final target of specific CD4 + T cells and antibodies in both primary infection and vaccination induced [10]. Different studies have demonstrated how traces of *herpesviridae* (and VZV) may persist for years in previously infected tissues suggesting that these residual particles may trigger site-specific inflammatory reactions in hosts with an up-regulated immunity after vaccination [27–30].

The second mechanism involves a non-specific hyperactivation of the immune system, possibly triggered by RZV. Lehmann et al. reported a case of multifocal choroiditis reactivation following RZV vaccination, supporting the theory that vaccines could dysregulate immune response in predisposed individuals [31]. Similarly, in 2019, Heydari-Kamjani et al. described a ‘presumed’ ocular sarcoidosis started shortly after RZV injection in a patient with no history of uveitis [32]. The authors suggested that RZV components may be the determinant factors, with particular concern for the two immunostimulants composing the novel adjuvant AS01B: MPL (3-O-desacyl-4′-monophosphoryl lipid A), derived from the *Salmonella Minnesota* lipopolysaccharide and the saponin derived QS21, extracted from the South American tree *Quillaja Saponaria Molina.* In combination, these two molecules proved to be highly effective stimulators of dendritic cells activation and antigen presentation to T cells [10, 32]. Moreover, in vivo experimental models in rats have shown that MPL can induce intraocular inflammation and increase ocular vascular permeability [33].

In our case the development of inflammatory findings only in the eye with resolved VZV infection suggests a VZV-specific inflammatory reaction, triggered by vaccination rather than a non-specific hyperactivation of the immune system which would have involved both eyes. On top of this, the absence of active foci of retinitis, as demonstrated by multimodal imaging (MMI) along with the rapid resolution of symptoms following steroid therapy initiation support the hypothesis of a purely reactive nature of the inflammation.

Interestingly, similarities can be found between our case and the well-known immune reconstitution inflammatory syndrome (IRIS), a condition observed in patients with acquired immunodeficiency syndrome (AIDS) and concurrent opportunistic infections, who are treated with highly active antiretroviral therapy (HAART). Elevation in CD4 + T cells count, following HAART, can result in an uncontrolled systemic and organ-specific inflammation [34]. Immune Recovery Uveitis (IRU) is a possible manifestation of IRIS commonly observed in patients with prior cytomegalovirus (CMV) retinitis and presents with varying degrees of intraocular inflammation, including anterior uveitis, vitritis, papillitis and CME, potentially leading to severe permanent visual impairment [35, 36].

We hypothesize that, similarly to IRU, our case of intraocular inflammation may be due to immune system reactivation following vaccination, leading to a breakdown of the blood-retinal barrier and to the development of CME. As in IRU, corticosteroid therapy was effective in reducing the inflammatory response. Further research could determine whether larger areas of retinitis in ARN could predispose to more frequent and severe intraocular inflammation after vaccination with RZV.

In conclusion, RZV remains a safe and effective measure against herpes zoster (HZ) and its complications. However, physicians and patients should be aware of the potential, albeit rare, local, and systemic inflammatory adverse effects that may follow vaccination, including intraocular inflammation. The observed reaction may imply a pathogen-specific response, bearing similarities to Immune Recovery Uveitis (IRU) in both pathogenesis and clinical presentation. Further research is needed to establish the incidence and risk factors associated with these events.

## Data Availability

No datasets were generated or analysed during the current study.

## Abbreviations

APMPPE: Acute posterior multifocal placoid pigment epitheliopathy
ARN: Acute retinal necrosis
BRB: Blood-retinal barrier
CME: Cystoid macular edema
EMA: European Medicines Agency
FA: Fluorescein angiography
FAF: Fundus autofluorescence
gE: Glicoprotein E
HSV: Herpes Simplex Virus
ICGA: Indocyanine green angiography
MEWDS: Multiple evanescent white dot syndrome
NIR: Near-infrared reflectance
RZV: Recombinant varicella-zoster vaccine
SD-OCT: Spectral-domain optical coherence tomography
VZV: Varicella-Zoster Virus
VKH: Vogt-Koyanagi-Harada
